# Expression of glycoproteins with excellent glycosylation profile and serum half-life in CAP-Go cells

**DOI:** 10.1186/1753-6561-9-S9-P12

**Published:** 2015-12-14

**Authors:** Silke Wissing, Jens Wölfel, Helmut Kewes, Christian Niehus, Corinna Bialek, Sabine Hertel, Nicole Faust

**Affiliations:** 1 CEVEC Pharmaceuticals, 51105 Cologne, Germany, www.cevec.com

## Background

Due to their clinical importance, the development of therapeutic proteins has accelerated immensely over the past years. However, the expression of highly glycosylated recombinant therapeutic proteins, as for example blood coagulation factors or serum proteins has remained a challenging task. Human cell lines, as HEK293 cells or our amniocyte-derived CAP cell line, appear to be efficient in producing and secreting these proteins, however glycosylation can be incomplete.

Although, we have found that CAP cells generate a more authentic human glycosylation pattern than HEK293, we have for some proteins, e.g. human C1 esterase inhibitor (hC1-Inh), also detected incomplete sialylation, resulting in reduced serum half-life of the recombinant protein.

C1 esterase Inhibitor (C1 Inh) belongs to the serpin superfamily. Its main function is the inhibition of the complement system to prevent spontaneous activation. The 500 aa protein is highly glycosylated with 7 predicted N-glycans and 8 predicted O-linked glycans. Plasma derived C1 Inhibitors (Berinert, CSL Behring and Cinryze, ViroPharma,) as well as recombinant C1 Inh derived from milk of transgenic rabbits (Ruconest, Pharming N.V.) are approved for the treatment of acute attacks in patients with hereditary angioedema (HAE). However, the recombinant product shows a dramatically reduced serum half-life in pharmacokinetic studies in comparison to the plasma derived counterparts.

## Results

**Figure 1:**
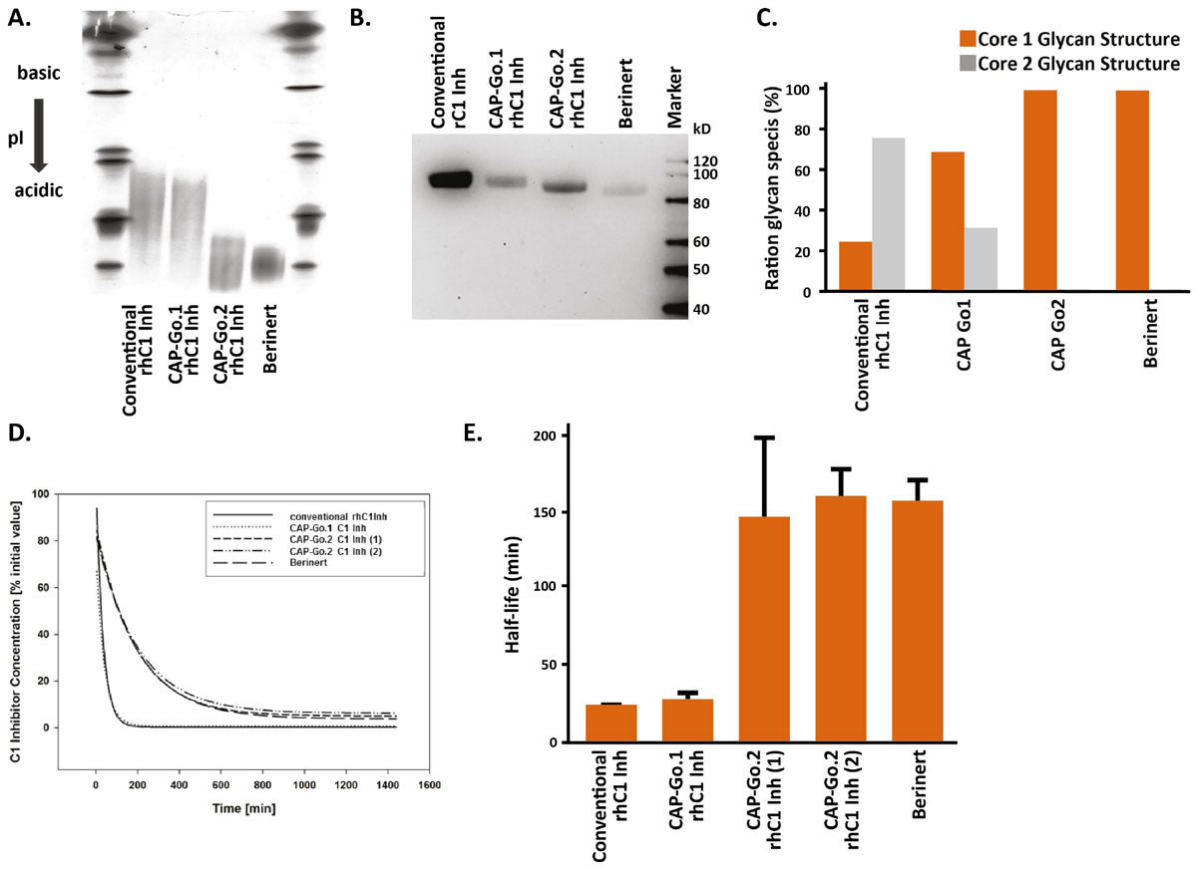
**Expression of recombinant human C1 Inh in CAP-Go**.2 cells results in glycoproteins with increased sialylation of N-glycans, homogenous core 1 O-glycosylation and significant increased serum half-life**. A) **Recombinant proteins were produced using the indicated platform, purified and subjected to isolelectric focusing (IEF) gel analysis in comparison to plasma-derived Berinert. **B) **ECL Lectin Blot, comparing degree of sialylation between different CAP-Go cell lines and hC1 Inh from human serum (Berinert). *Erythrina cristagalli *lectin binds to terminal galactose on N-glycans, sialic acid prevents the lectin from binding, therefore decrease signal intensity indicates increase in sialylation. **C) **MALDI-TOF mass spectrum analysis of C1 Inh O-glycans of protein expressed in CAP-Go.1 or CAP-Go.2 cell lines in comparison to Berinert. **D and E) **C1 Inh preparations were injected intravenously at 10 mg/kg into female Sprague Dawley rats. Residual C1 in plasma samples taken at the indicated time points was analyzed by ELISA.

We have further developed the CAP cell line (CAP-Go cells) to confer optimal glycosylation to highly complex glycoproteins like C1 Inh. Among these, the CAP-Go.1 cell line has been modified to facilitate expression of proteins with fully sialylated N-linked glycans. Recombinant proteins like human alpha-antitrypsin or human placental alkaline phosphatase produced with CAP-Go.1 show a significantly prolonged serum half-life in rats (data not shown). However, when human C1 Inh is expressed in CAP-Go.1 cells, this has no positive impact on the pharmacokinetic profile (Figure 1, D+E). Additional modifications of the CAP cell line have resulted in the CAP-Go.2 cell line, which addresses the O-linked glycosylation patterns.

Expression of recombinant human rhC1Inh in CAP-Go.2 cells results in a significantly increased serum half-life of the produced protein compared to its counterpart generated on a conventional expression platform and is actually indistinguishable from to the plasma-purified protein (Figure 1, D+E). While rhC1 Inh proteins expressed in either CAP-Go.1 or CAP-Go.2 cells both show a similar reduction of free terminal galactose on N-linked glycans (Figure 1, B), their O-glycans differ. Analysis of the O-Glycans shows that rhC1 Inh expressed by CAP-Go.2 cells, but not CAP-Go.1, contains only core1 O-linked glycan structures, highly comparable to plasma-derived Berinert. Overall, glycans of recombinant C1 Inh expressed in CAP-Go.2 cells are more homogenous than the glycans found on conventional recombinant glycoproteins, resulting in lower batch to batch variations and reduced downstream cost due to high expression of recombinant proteins with the desired glycoprofile.

## Conclusions

In this work, we present our approach for the expression of recombinant glycoproteins, like human C1 Inhibitor (rhC1 Inh) with excellent pharmacokinetik properties using modified CAP cells. Our results indicate that in addition to N-glycosylation, also the structure of O-linked glycans plays a crucial role in bioavailability and pharmacokinetic properties of glycoproteins. rhC1 Inh expressed from CAP-Go.2 cells that have been optimized for the expression of N- and O-glycosylated proteins display glycan patterns closely similar to plasma-derived C1 Inh and the resulting molecule has a significantly prolonged serum half-life as compared to its counterpart generated on a conventional human cell line. Our new recombinant molecule matches serum-derived C1 in all aspects analyzed: specific activity, serum half-life, and glycosylation pattern (Figure 1 and data not shown) and offers the advantage of being producible at large scale on a safe platform.

Glycans found on non-antibody biopharmaceuticals can be highly complex involving antennary fucose, Lac-di-Nac structures and a variable number of antennae, just on N-linked glycans. To address these challenges we have developed additional CAP-Go cell lines that can be employed to generate recombinant proteins with tailor made glycan structures.

